# Childhood Acute Poisoning at Haiphong Children's Hospital: A 10-Year Retrospective Study

**DOI:** 10.1155/2023/2130755

**Published:** 2023-09-04

**Authors:** Sang Ngoc Nguyen, Lam Tung Vu, Ha Thai Nguyen, Le My Thi Nguyen

**Affiliations:** ^1^Haiphong University of Medicine and Pharmacy, Haiphong, Vietnam; ^2^Hanoi Medical University, Hanoi, Vietnam; ^3^Haiphong Children's Hospital, Haiphong, Vietnam

## Abstract

**Introduction:**

Children are most often harmed by acute poisoning, which may cause disability or even death. This demonstrates the critical necessity for epidemiologic studies specific to each nation and area since they aid in developing plans for the prevention of acute poisoning. There are no data or outdated data on acute poisoning in children in Vietnam. This research would partly fill this existing gap and compare the trend with other places across the globe.

**Methods:**

A retrospective study was conducted in the 10-year period from 2012 to 2021 in Haiphong Children's Hospital, Vietnam.

**Results:**

There were 771 children hospitalized due to acute poisoning. Children in the 1–5-year-old group accounted for the highest rate, at 506 (65.6%). The mean age was 4.5 ± 4.1 years old. The male-to-female ratio was 1.2/1. Nonpharmaceutical chemicals were the most common agent in 331 cases (42.9%), including cleaning products 63 (19.0%), rat poison 60 (18.1%), and petrol 42 (12.7%). Medications were the second most common agent in 290 cases (37.6%), mostly paracetamol 60 (20.7%) and sedatives 40 (13.8%). There were 633 (82.1%) children exposed to poisons unintentionally.

**Conclusion:**

Children between the ages of 1 and 5 are more likely to be exposed to harmful substances. The most common agents were nonpharmaceutical chemicals followed by pharmaceuticals. Most incidents were inadvertent. Finally, our research may provide insights that public health authorities might use to plan practical actions.

## 1. Introduction

The house and its surroundings can be dangerous to children, especially when it comes to the risk of accidental poisoning. Children are naturally interested and enjoy exploring their surroundings. As a result, acute poisoning in children remains a major public health concern that requires special attention, particularly in hospital emergency departments. Acute poisoning has become the primary cause of harm to children, resulting in disability and death [1]. According to a report published by the World Health Organization (WHO) in 2008 [2], an estimated 45,000 deaths among children and young people (under the age of 20) are caused by acute poisoning each year. Low-income nations have a four-fold greater mortality rate than developed countries [3]. Many severe poisoning cases in children have occurred in recent years, owing to parents' careless storage of chemicals or medicine and others in kindergarten, nursery school, and other places. In addition, parents purchasing medication for their children without a doctor's prescription resulted in an increase of acute poisoning by medication [4].

In recent years, acute poisoning patterns have changed from country to country [5, 6]. Therefore, to lower the incidence of acute poisoning and mortality in children, the causes, frequency, and circumstances of poisoning, as well as the factors that influence the death rate caused by acute poisoning, must be analyzed in different countries and regions. This reveals the great need for epidemiologic studies that are specific for each country and region as this helps to plan for acute poisoning prevention. The current study acquired clinical data from 771 children admitted to Haiphong Children's Hospital in Vietnam between January 01^st^, 2012, and December 31^st^, 2021. The data were systematically retrospectively analyzed to investigate clinical, epidemiological characteristics of childhood acute poisoning.

## 2. Materials and Methods

A retrospective study was conducted for a 10-year period, from January 01^st^, 2012, to December 31^st^, 2021. The study site was Haiphong Children's Hospital in Haiphong City, Vietnam.

### 2.1. Inclusion Criteria

All patients aged 0 to 15 years were diagnosed with acute poisoning using the diagnostic criteria for acute poisoning, consistent with an expert consensus and the International Classification of Diseases (ICD) 10^th^ revision.

### 2.2. Exclusion Criteria

Clinical symptoms and signs were suspected of acute poisoning but lacked evidence for diagnosis.

### 2.3. Methodology

Individual patient information and data were obtained from the Haiphong Children's Hospital documentation database. The data collected includes personal and demographic information (age, sex), date and time of admission, number (one or more) and type of substance involved, route of exposure, previous episodes of poisoning, mode of poisoning (suicide attempt, intentional recreational, accidental, and therapeutic error), and possible action of caretakers before arrival at the emergency room (cleansing, induction of vomiting, and giving of food, water, or milk). There was no further investigation.

The substance was identified based on the patient's whole history (as stated by the poisoned patients and their caregivers) and/or a sample or bottle of the poison shown to the physician by the patients and their caregivers.

#### 2.3.1. The Substance Classification

1. Nonpharmaceutical chemicals including cleaning products, rat poison, petrol, and pesticide
2. Pharmaceuticals/medications
3. Narcotics
4. Foodstuffs including puffer fish and mushrooms
5. Animal-related injuries including snakebite, beesting, and insect bites

### 2.4. Data Analysis

The data were analyzed by Statistical Package for Social Sciences (SPSS) software version 26.0 and Microsoft Excel 365. All data are presented as mean ± standard error of the mean. To describe the continuous and qualitative variables, mean, standard deviation, and frequency (%) were reported.

### 2.5. Ethical Approval

Approval of the study was obtained from the Medical Ethics Council of Haiphong University of Medicine and Pharmacy (number 224/QD-YDHP, 21/01/2022). As this was a retrospective study, informed consent was waived by the Medical Ethics Council.

## 3. Results

**Figure 1:**
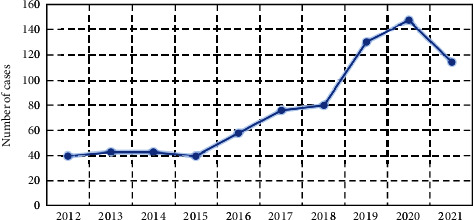
The number of children hospitalized due to childhood acute poisoning each year.

During the 10 years from January 1st, 2012, to December 31st, 2021, there were a total of 771 children (426 males and 345 females) hospitalized at Haiphong Children's Hospital with the diagnosis of acute poisoning. The number of acute poisoning cases increased dramatically during the period from 2018 to 2020 (see Figure 1). The mean age was 4.5 ± 3.6 years old. The male-to-female ratio was 1.23. The number of children arriving from the suburbs (415 cases) was greater than those from the city (356 cases) (Table 1). Most cases had the time between the event and arrival at the hospital less than 6 hours, accounting for 90.4%.

There were five primary agent groups causing acute poisoning. Of those, nonpharmaceutical chemicals were highest with 42.9% of exposures, followed by medications (37.6%). In the chemical group, the most frequent agents were cleaning products, accounting for 19.0%, followed by rat poison (18.1%) and petrol (12.7%). Meanwhile, in the medication group, paracetamol was the leading cause, with 60 cases (20.7%), followed by sedatives (40 cases, 13.8%) (see Table 2).

Table 3 indicates that unintentional poisoning occurred more commonly in children aged 1 to 5 (68.9%), whereas the proportion of intentional poisoning was the greatest in the age range from 11 to 15 years old (89.2%). Males were more likely than females to be poisoned unintentionally, while the number of intentional cases in females was higher than in males.

## 4. Discussion

Acute poisoning in pediatrics has been recognized as a common cause of admission to emergency departments globally, yet, data on this expected public medical issue is limited. This highlights the crucial need for epidemiologic studies tailored to each country and region since they contribute to the establishment of programs for the prevention of acute poisoning.

The frequency of acute poisoning in children fluctuated over the period. In 10 years, from January 1^st^, 2012, to December 31^st^, 2021, we noted that 771 cases were hospitalized at Haiphong Children's Hospital. As Figure 1 shows, the number of cases peaked in 2020. At that time, Vietnam was experiencing Vietnam's social distancing law during the COVID-19 pandemic, which forced all residents to isolate socially and forced an increasing number of children to be kept at home rather than attending school. As a consequence, children were more likely to come into contact with dangerous substances.

Acute poisoning may appear at any age. The youngest participant in our research was 28 days old, who was exposed because he was administered a nasal spray containing naphazoline without a doctor's prescription; and the oldest was 15 years old. Our result regarding the average age was much less than Đặng and Đỗ's study in Bach Mai Hospital, Vietnam, which was 14.4 ± 3.9 years old [4]. The difference with the study of Đặng and Đỗ is due to the fact that they include patients < 18 years old and declare that the vast majority were adolescents. Meanwhile, a study in Iran about the epidemiological and clinical features of acute poisoning in children showed that the average age of the patients was 3.4 years [7].

Acute poisoning occurred in both males and females. In our study, the male-to-female ratio was 1.23/1 (male: 55.3% and female: 44.8%). Similar findings have been reported by Nguyen et al. (male: 60.2% and female: 39.8%) [8], AAPCC (male: 51.9% and female: 48.1%) [9], and Pawlowicz et al. (male: 52.4% and female: 47.6%) [10].

Our research found that children from cities (54%) had a greater poisoning incidence than those from suburbs (46%), which can be explained by the fact that childhood poisoning was not always severe, and because of the great distance from their homes to Haiphong Children's Hospital, parents did not take their children to our hospital for treatment and treated at local hospitals, instead. Hamid et al. also noted the same findings as our results that 80% of children with acute poisoning lived in urban areas [11].

The interval between the moment of poisoning and admittance to the hospital was a major determinant in physicians' ability to provide the correct treatment. In our study, interval of our patients was mostly less than 6 hours (90.4%). Nguyen et al. noted that 61.6% of patients had an interval between the moment of poisoning and admittance to the hospital of less than 6 hours [8]. Chatterjee et al. reported that the median time between the event and arrival at the hospital was 11 hours [12].

We found that nonpharmaceutical chemicals were the most common causative agent, with 331 cases accounting for 42.9%, followed by medications (37.6%). Đặng and Đỗ also found the same findings that nonpharmaceutical chemicals were the most common agent causing poison in children hospitalized in Bach Mai Hospital, Vietnam at 40% [4]. Unlike our results, Gkentzi et al. showed that most cases were due to the ingestion of tobacco-related products in Greece [13], and Chatterjee et al. noted that snakebite was the most common in Indian children [12]. The differences in the geographical factors can explain the differences in the results of those studies. Greek children may be exposed to poison due to their parents' smoking habits, while the environment in which Indian children live may be snake-infested. Moreover, chemical compounds were the most widespread in Vietnam due to a lack of childcare at home, resulting in accidental ingestion of chemical items.

In terms of chemical exposure causing acute poisoning in children, cleaning products rank first in our study, with 63 out of 331 cases (19.0%), followed by rat poison (18.1%) (see Table 2). Similar findings were also noticed by Koueta et al., whose research showed that 44.7% of patients hospitalized related to household cleaning liquids [14]. In the world, detergents were one of the common causative agents leading to acute poisoning, which was observed by Gontko et al. (50%), Berta et al. (49%), Hahn et al. (46%), and Azkugana et al. (24.4%) [15–18]. Parents accidentally left these substances within reach of their children, which resulted in the children's ingestion of those chemicals. Moreover, rat poison and insecticide were the other nonpharmaceutical chemicals that led to some severe acute poisoning cases. Several articles noted that rat poison and insecticide were the leading cause of acute poisoning, especially in agricultural countries such as India (16%) and Bangladesh (27.6%) [5, 19]. The third common chemical in our study was petroleum products, accounting for 12.7%. However, these nonpharmaceutical chemicals take the first place on the list of hazardous substances causing poisoning in children, according to the research of Koueta et al. [14] and Jayashree and Singhi [19]. Hamid et al. also pointed out that 23% of the patients included in the study were admitted to the hospital due to petrol [11]. All the findings demonstrated the problem of negligent parents placing petroleum in commonly used bottles, perhaps causing children to consume it accidentally.

Regarding acute poisoning due to medication, we found 290 cases in the ten years (see Table 2). Our findings indicated that paracetamol was the leading cause in this group, accounting for 20.7%. Many studies in the world showed the same results as ours. Tobaiqy et al. noted that 39.1% of the patients included in the study were admitted because of paracetamol, taking first place in the medication group [20]. According to Gkentzi et al., 25% of children hospitalized due to drug consumption were due to paracetamol [13]. Moreover, Dayasiri et al. pointed out that paracetamol ranked second [21]. However, Koueta et al. noted that sedatives were the most common drug causing poisoning in children (46.4%), while they only contributed 13.8% to our study [14]. Chowdhury et al. also had the same findings, with 13.4% of poisoning patients caused by sedatives [5].

We also found 24 cases (3.1%) of being exposed to narcotics accidentally. All the cases had at least one of their family members who was a drug addict. Đặng and Đỗ conducted a study in Bach Mai Hospital, Vietnam, and also had the same result, with 4% being due to narcotics [4].

In terms of animal injury, we only discovered 19 incidents (2.5%) over ten years, with one case related to a beesting and 18 cases due to venomous snakes. This finding is much lower than those observed by Đặng and Đỗ (poisoning due to animals: 27.0%) [4]. This might be explained by the great number of poisonous snakes and bees in Hanoi and the surrounding areas, which resulted in a high incidence of poisonings.

The majority of acute poisoning cases were unintentional (82.1%). This finding was the same as that of Sahin et al., which showed that 73.3% of all patients had accidental poisoning [22]. In addition, several authors also indicated that most acute poisonings were unintentional, such as Song et al. [23] and Azab et al. [24]. In Vietnam, Nguyen et al. also noted that 91.8% of children hospitalized in Bach Mai Hospital due to poisoning were accidental [8]. Most of the cases were aged from 1 to 5 years old (68.9%). Children at this age are curious, with a natural desire to investigate their surroundings and a lack of knowledge of potential dangers. Consequently, they will put everything in their grasp, particularly colorful items such as medications or chemicals, into their mouths.

Furthermore, one of the key contributors to this circumstance may be the negligence of parents and child caregivers, who kept chemicals or prescriptions in readily accessible areas within reach of the kid or administered them in unlabeled or wrongly labeled containers. For example, petroleum was regularly kept in bottles used for fruit juice, resulting in children accidentally consuming it. Therefore, parents and guardians must be responsible for the proper storage of medications and home-cleaning chemicals, as well as teaching children about the dangers of poisoning [24, 25].

In terms of intentional poisoning (37 cases—12.6%), all occurrences were suicide attempts and occurred after age 6, with the majority occurring between the ages of 11 and 15, accounting for 89.2%. Hahn et al. also found that 20% of the patients included in the study were suicide attempts [17]. Our study also showed the same pattern as Li et al.'s study, which found that 16.8% of Chinese children were suicide attempts [26]. In our study, the number of cases that appeared in females was more than in males (Table 3). Our finding was consistent with the result of Đặng and Đỗ [4] and Li et al. [26]. This age group is transitioning from childhood to adulthood and has significant psychophysiological differences, particularly in girls. As a result, they are easily hurt psychologically. They react passively when parents or teachers keep shouting at them, criticizing them for poor academic performance, or have conflicts with friends or families. Poisoning suicides among teenage females could be related to an awakening of their sense of self, unstable emotions, increasing pressure from academics, society, and even sexual interactions [27]. Meanwhile, females are more emotionally rich and less resistant to irritation than males [28].

In the group of incorrect usage of medication, there were 37 cases (4.8%). Parents lacked sufficient understanding of all medicine types, including the proper dosage, indications, and contraindications. All individuals in this group had a history of receiving excessive amounts of prescription medication from their parents.

The study's main limitation was that it was solely retrospective work. Furthermore, since many patients remain at home, this research cannot catch nonhospitalized poisoning. Although our research indeed focuses on acute poisoning in children in Haiphong City, it does not represent Vietnam as a whole.

## 5. Conclusions

Childhood acute poisoning is still an avoidable public health issue worldwide, especially in Haiphong, Vietnam. Children between the ages of 1 and 5 need more attention from parents because they are more likely to be exposed to harmful substances. The most common agents were nonpharmaceutical chemicals (cleaning supplies and rat poison), followed by pharmaceuticals (paracetamol and sedatives). Most incidents were inadvertent. Finally, our research may provide insights that public health authorities might use to plan effective actions.

## Figures and Tables

**Table 1:** The demographic of admitted patients (*n* = 771).

	Number of patients (*n*)	Percentage (%)
*Age*		
<1 years	83	10.8
1–5 years	506	65.6
6–10 years	101	13.1
11–15 years	81	10.5
*Sex*		
Male	426	55.3
Female	345	44.7
*Geographical distribution*		
Urban	356	46.2
Rural	415	53.8
*Time elapsed before admission*		
<360 mins	697	90.4
≥360 mins	74	9.6

**Table 2:** Poisoning agent distribution in children with acute poisoning (*n* = 771).

Agent	Number of cases (*n*)	Percentage (%)
*Nonpharmaceutical chemicals*	331	42.9
Cleaning products	63	19.0
Rat poison	60	18.1
Petrol	42	12.7
Others	166	50.2
*Medications*	290	37.6
Paracetamol	60	20.7
Sedatives	40	13.8
Others	190	65.5
*Foodstuffs*	107	13.9
*Narcotics*	24	3.1
*Animal-related injuries*	19	2.5
Total	771	100

**Table 3:** Mode of poisoning according to age and sex of intoxicated children (*n* = 771).

	Age (*n*, %)				Sex (*n*, %)		Total
	<1 year	1–5 years	6–10 years	>10 years	Males	Females	
Unintentional	69 (10.9)	436 (68.9)	89 (14.1)	39 (6.1)	352 (55.6)	281 (44.4)	633
Intentional	0 (0)	0 (0)	4 (10.8)	33 (89.2)	16 (43.2)	21 (56.8)	37
Incorrect usage	14 (14.4)	68 (70.1)	7 (7.2)	8 (8.3)	56 (57.7)	41 (42.3)	97
Homicidal	0	2 (50.0)	1 (25.0)	1 (25.0)	2 (50.0)	2 (50.0)	4

## Data Availability

Data and materials used and/or analyzed during the current study are available from the corresponding author upon reasonable request.
